# Identification of a nomogram based on an 8-lncRNA signature as a novel diagnostic biomarker for childhood acute lymphoblastic leukemia

**DOI:** 10.18632/aging.203116

**Published:** 2021-06-09

**Authors:** Zhang Chen, Fan Yang, Hui Liu, Fan Fan, Yanggang Lin, Jinhua Zhou, Yun Cai, Xiaoxiao Zhang, Yingxin Wu, Rui Mao, Tongtong Zhang

**Affiliations:** 1 Affiliated Hospital of Southwest Jiaotong University, Chengdu 610036, China; 2 Emergency Department, Peking University Third Hospital, Peking University School of Medicine, Beijing 100083, China; 3 Department of Neurology, General Hospital of Western Theater Command, Chengdu 610500, China; 4 Department of Orthopedics, General Hospital of Western Theater Command, Chengdu 610083, China; 5 West China School of Public Health and West China Fourth Hospital, Sichuan University, Chengdu 610041, China; 6 Center of Gastrointestinal and Minimally Invasive Surgery, Department of General Surgery, The Third People’s Hospital of Chengdu, Affiliated Hospital of Southwest Jiaotong University and The Second Affiliated Hospital of Chengdu, Chongqing Medical University, Chengdu 610031, China; 7 Medical Research Center, The Third People's Hospital of Chengdu, The Second Chengdu Hospital Affiliated to Chongqing Medical University, Chengdu 610031, China

**Keywords:** childhood acute lymphoblastic leukemia, prognosis, nomogram, LINC01278, hsa-miR-500b

## Abstract

Childhood acute lymphoblastic leukemia (cALL) still represents a major cause of disease-related death in children. This study aimed to explore the prognostic value of long non-coding RNAs (lncRNAs) in cALL. We downloaded lncRNA expression profiles from the TARGET and GEO databases. Univariate, least absolute shrinkage and selection operator (LASSO) and multivariate Cox regression analyses were applied to identify lncRNA-based signatures. We identified an eight-lncRNA signature (LINC00630, HDAC2-AS2, LINC01278, AL356599.1, AC114490.1, AL132639.3, FUT8.AS1, and TTC28.AS1), which separated the patients into two groups with significantly different overall survival rates. A nomogram based on the signature, BCR ABL1 status and white blood cell count at diagnosis was developed and showed good accuracy for predicting the 3-, 5- and 7-year survival probability of cALL patients. The C-index values of the nomogram in the training and internal validation set reached 0.8 (95% CI, 0.757 to 0.843) and 0.806 (95% CI, 0.728 to 0.884), respectively. The nomogram proposed in this study objectively and accurately predicted the prognosis of cALL. *In vitro* experiments suggested that LINC01278 promoted the proliferation of leukemic cells and inhibited leukemic cell apoptosis by targeting the inhibition of miR-500b-3p in cALL, and LINC01278 may be a biological target for the treatment of cALL in the future.

## INTRODUCTION

Acute leukemia is the most common childhood malignant tumor. And there would be one patient in every three children who has it in the developed countries and nearly 50% in developing countries [1]. The commonest leukemia is subtype B-lineage acute lymphoblastic leukemia (B-ALL) [2, 3]. The survival rate of patients who have childhood acute lymphoblastic leukemia (cALL) is good. However, relapse of children who have subsets of cALL is in in high rate, especially for infants, and the cure rate is not desired [4]. Therefore, better clinical and biological prognosis stratification is mandatory before treatment initiation.

Long non-coding RNAs (lncRNAs) are RNAs with a length of more than 200 nucleotides [5]. Although lncRNAs are not involved in protein-coding, there is growing evidence that they take part in the biological processes of cell proliferation, apoptosis and differentiation [6, 7]. Sufficient information indicates that lncRNAs are involved in tumor progression and the regulation of tumor biological behavior through interaction with microRNAs (miRNAs) or messenger RNAs (mRNAs) [8–11]. Many well-known lncRNAs are treated as diagnostic or prognostic biomarkers for ALL, such as ARIEL [12], IUR [13], CASC15 [14], and BALR-6 [15]. TARGET is a dynamically updated data improving base of (OCG), NCI's Office of Cancer Genomics, whose goal is to promote molecular understanding of cancer for the prognosis of patients. cALL is a project in the TARGET plan, including the first phase (B-ALL), the second phase (B-ALL, T-ALL) and the third phase (ALL) sample. The samples with complete clinical information in phase II are used, which included 426 B-ALL patients.

cALL is a complex disease composed of many subtypes with unique somatic genetic changes, including aneuploidy, chromosomal rearrangements, and point mutations [16]. To date, growing evidence has shown that lncRNAs are involved in many pathophysiological processes in the progress of tumor and are the link between genes and tumorigenesis [17, 18]. Fernando et al. [19] for the first time observed in patients with cALL that the lncRNA BALR-2 correlates with overall survival (OS) and with response to prednisone. The research team also confirmed that the overexpressed lncRNA CASC15 in RUNX1-rearranged cALL patients affects their prognosis by upregulating the chromosomally adjacent gene SOX4 [14]. In another study, Garitano-Trojaola et al. [20] found 43 abnormally expressed lncRNAs by comparing the peripheral blood samples of normal and cALL patients. Among them, LINC-PINT was significantly downregulated in T and B-ALL, and tumor cell growth could be inhibited by restoring its expression. These studies suggest that lncRNAs might be used as diagnostic and prognostic markers for cALL.

To identify lncRNA associated with prognosis and guide clinical applications in cALL, we combined RNA-seq data and clinical information from 426 B-ALL patients to create a nomogram with an 8-lncRNA signature.

## RESULTS

### Preprocessing of the datasets

A matrix was downloaded from the TARGET database. 532 participants in total with cALL had lncRNA probe expression; after excluding 76 peripheral blood sources and 30 patients older than 15 years, 426 bone marrow-derived patients remained. Patients with cALL in the TARGET cohorts (n=426) were separated into training (n=300) and validation (n=126) sets, and their clinical features are presented in Table 1. The cohort from GSE34861 contains 194 ALL patients. After excluding 10 patients without survival time or status, a total of 184 samples remained. Matrix data were annotated based on GENCODE (Release 29), and 19754 mRNAs and 14847 lncRNAs were obtained in the TARGET cohort. After a two-step screening of probes and deleting those with zero expression and those with a standard deviation less than 1.2, we finally obtained 1280 lncRNAs and 4985 mRNAs.

**Table 1:** The clinical information of patients in the training dataset, internal validation dataset and entire validation dataset.

**Characteristics**	**Training dataset** **target-ALL (n=300)**	**Validation dataset** **target-ALL (n=126)**	**Validation dataset** **target-ALL (n=426)**
**Age (y)**			
<10	171	80	251
≥10	129	46	175
**Gender**			
Male	203	81	284
Female	97	45	142
**Survival status**			
Alive	223	98	321
Dead	77	28	105
**CNS Status**			
CNS1	222	95	317
CNS2	62	25	87
CNS3	16	6	22
**BCR ABL1 Status**			
positive	2	3	5
negative	298	123	421
**BMA Blasts Day 8**			
>20%	166	71	237
≤20%	134	55	189
**BMA Blasts Day 29**			
>5%	6	3	9
≤5%	294	123	417
**WBC (x10^3^/mcL)**			
<100	92	37	129
100 to 200	50	23	73
200 to 300	93	38	131
>300	65	28	93
**MRD of 29 days**			
≥1%	37	12	49
<1%	263	114	377
**DNA Index**			
1 to 1.16	278	115	393
>1.16	22	11	33

Abbreviations: WBC counts, WBC count at diagnosis; CNS Status, CNS status at diagnosis.

### Using 8-lncRNA markers to assess prognosis

**Figure 1:**
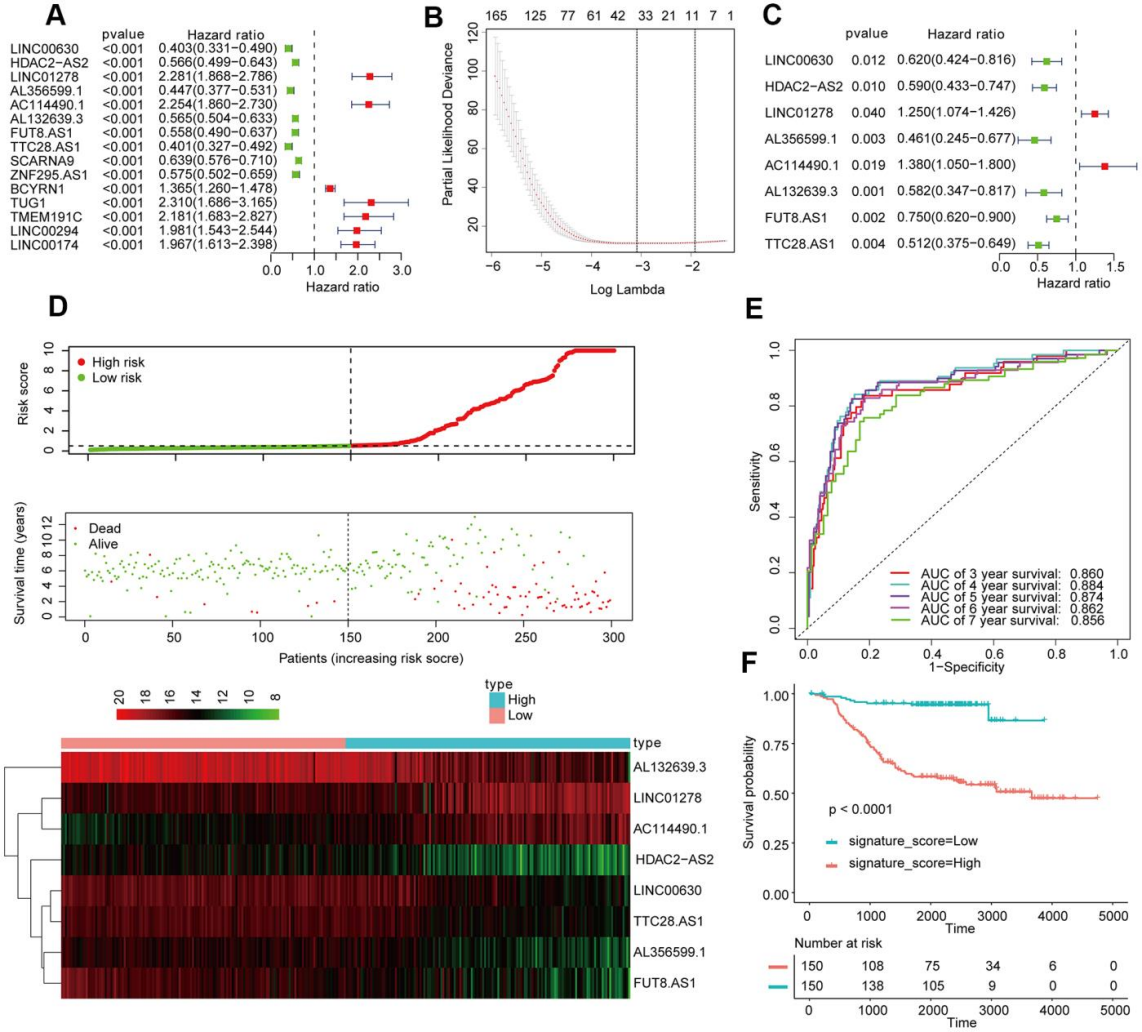
**Establishment and validation of the eight-lncRNA prognostic signature.** (**A**–**C**) The procedure for establishing the prognostic signature. (**D**) Correlation between the prognostic signature and the overall survival of patients in the TCGA cohort. The distribution of signature scores (upper), survival time (middle) and lncRNA expression levels (lower). The black dotted lines represent the median signature score cutoff dividing patients into low- and high-signature score groups. The red dots and lines represent the patients in the high-score groups. The green dots and lines represent the patients in the low-score groups. (**E**) ROC curve analyses based on the 8-lncRNA signature. (**F**) Kaplan-Meier curves of OS based on the 8-lncRNA signature.

A total of 1280 lncRNAs were applied to univariate Cox analysis, and 165 lncRNAs with P < 0.01 were filtered out and applied to subsequent analysis (Figure 1A). Then, 8 LncRNA (λ = 8) were screened out by lasso regression analysis, and multivariate Cox regression analysis was carried out on the 8 lncRNA (Figure 1B, 1C). In the end, 8 lncRNAs for prediction figured out: LINC00630, HDAC2-AS2, LINC01278, AL356599.1, AC114490.1, AL132639.3, FUT8-AS1, and TTC28-AS1. The signature score of these 8 lncRNAs was counted out according a formula: signature score = (0.22636 × exp of LINC01278) - (0.26743 × exp of LINC00630) - (0.21030 × exp of HDAC2-AS2) - (0.20700 × exp of AL356599.1) + (0.32054 × exp of AC114490.1) - (0.23411 × exp of AL132639.3) - (0.29040 × exp of FUT8.AS1) - (0.29665 × exp of TTC28.AS1). The cutoff point was the median signature score, participants in the training set were separated into a low-signature score group and a high signature-score group (Figure 1D). Participants with low-signature score were obviously better OS than patients with high-signature scores (Figure 1E). Also, ROC curve analysis compared whether prediction for survival was accurate in the 8_lncRNA-based signature (Figure 1F). The AUC values were assessed for 3-year (AUC = 0.860), 5-year (AUC = 0.874), and 7-year (AUC=0.856) survival.

### Construction of prognostic prediction nomogram according the 8-lncRNA signature and clinical characteristic

**Figure 2:**
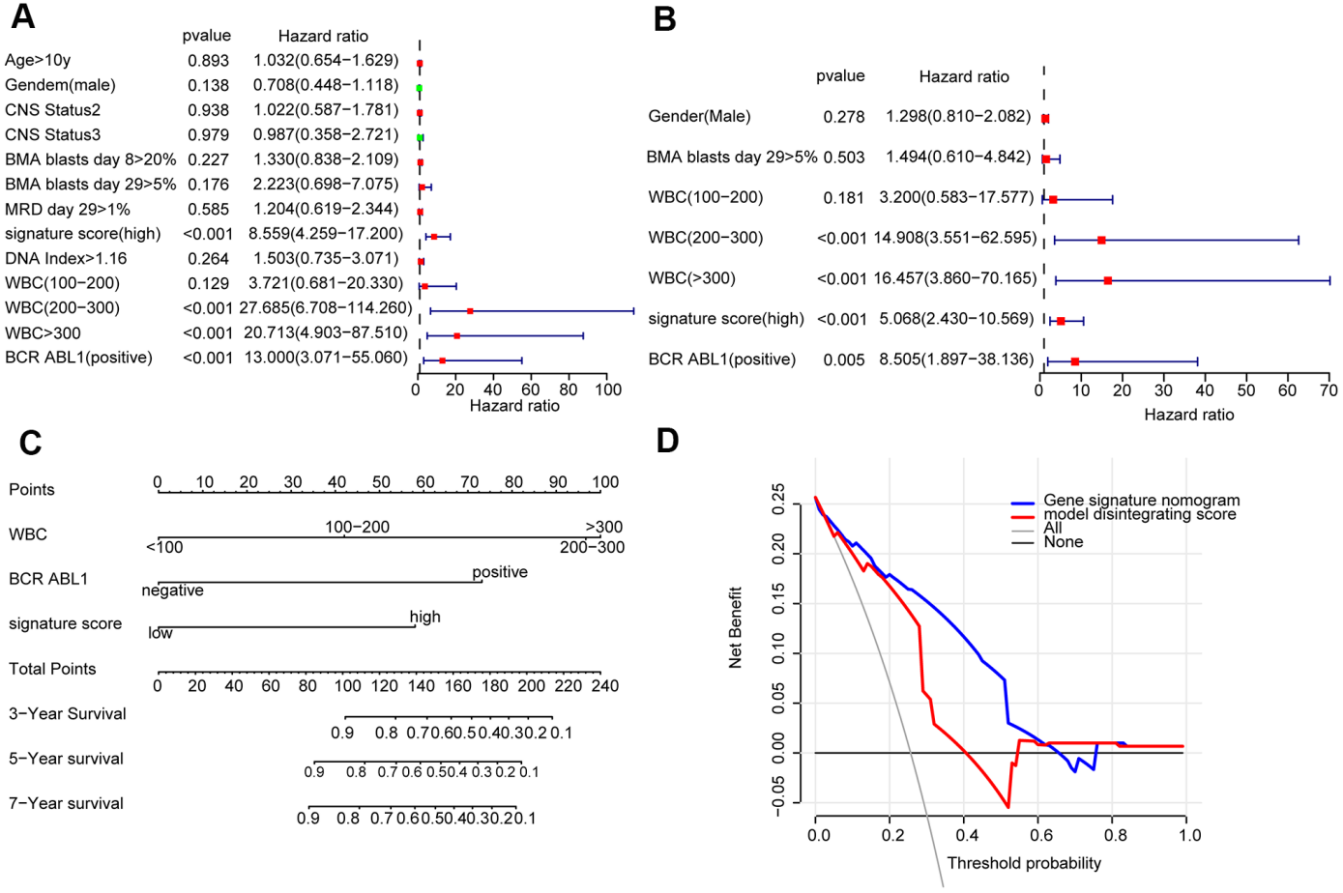
Construction of a nomogram for overall survival prediction in cALL (**A**, **B**). Univariate and multivariate Cox regression analyses of clinical factors associated with overall survival. (**C**). The nomogram consists of BCR ABL1 status, WBC count at diagnosis and the signature score. (**D**) Decision curve analysis for the cALL nomogram.

The prognostic variables were determined by univariate Cox regression analysis, and the candidate variables with P < 0.2 in univariate analysis were contained in multivariate COX regression analysis. (Figure 2A). Next, the multivariate Cox regression model screened out the following prognostic predictors: BCR ABL1 status; white blood cell (WBC) count at diagnosis; and signature score (Figure 2B). The nomogram is established by using the regression coefficients of the statistically significant variables in the multivariate Cox regression model. (Figure 2C). Surprisingly, the C-index of the nomogram reached 0.8 (95% CI, 0.757 to 0.843). The decision curve in training cohort showed that suppose the threshold possible of a patient and a doctor was >1% and <67%, respectively, the function the mentioned nomogram to predict prognosis of cALL would be more benefit than the scheme. It also indicated that the clinical benefit rate of the model containing the signature score was significantly higher than that without it (Figure 2D).

**Figure 3:**
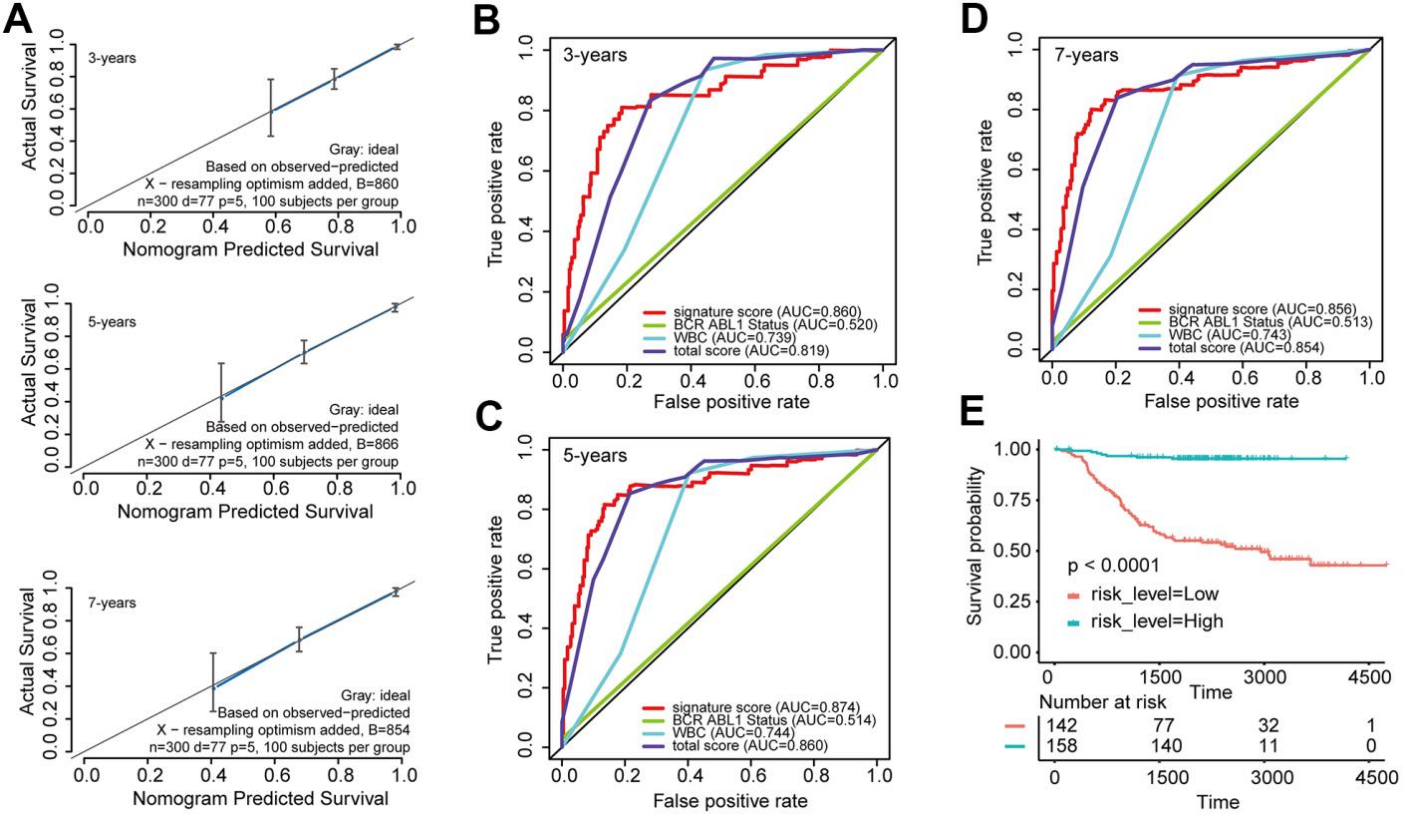
Evaluated the predictive accuracy and discriminative ability of the nomogram (**A**) Calibration curves of the nomogram for the estimation of survival rates at 3, 5, and 7 years. (**B**–**D**) ROC curves at 3, 5, and 7 years according to the nomogram and lncRNA signature score. (**E**) Kaplan-Meier curves of OS according to the total risk score.

Furthermore, the predictive values in the calibration curve were consistent with observed values when thinking the probabilities of 3-year, 5-year, and 7-year OS (Figure 3A). The AUC values for 3-, 5-, and 7-year survival reached 0.819, 0.860, and 0.854, respectively (Figure 3B–3D). In the end, the total risk score was counted out according to the risk proportion of each predictor in the nomogram. Kaplan-Meier analysis indicated that the OS of patients with low risk score was vitally better than that of those with a high risk score. (Figure 3E).

We evaluated BCR ABL1 fusion gene status and WBC at diagnosis by Kaplan-Meier analysis. In general, the subtype of patients who were positive for the BCR ABL1 fusion gene indicated worse OS than those who were negative for the BCR ABL1 fusion gene (Supplementary Figure 1A), and patients in the high-signature group had worse OS than those in the low- signature group in BCR ABL1 fusion negative segment (Supplementary Figure 1B). While in the BCR ABL1 fusion gene positive segment, all participants were in the high-signature group (Supplementary Figure 1C). Eventually, Kaplan-Meier analysis suggested that patients with a WBC count greater than 200 x10^3^/mcL at the first diagnosis have significantly worse OS than those with a WBC count less than 200 x10^3^/mcL (log-rank test P-value <0.0001, Supplementary Figure 1D).

### Validation of the 8-lncRNA signature for prognostic evaluation

To validate the robustness of the nomogram, the similar analysis process in the validation set was conducted. According to the signature score formula in the internal validation set (n =126), patients were separated into groups of those with a high-signature score (n = 63) and a low-signature score (n = 63) with the median signature score as the cutoff point (Supplementary Figure 2A). The Kaplan-Meier curves of OS shows that patients with low-signature scores have substantially better OS than patients with higher signature scores (Supplementary Figure 2B). The AUC exhibited by the 8-lncRNA signature for 3-, 5-, and 7-year survival reached 0.909, 0.851, and 0.813, respectively (Supplementary Figure 2C). Next, the nomogram model was built by using the coefficients of the multivariate Cox regression model. The C-index of the nomogram achieved 0.806 (95% CI, 0.728 to 0.884). The calibration curve indicated that predictive values were consistent with observed values considering the probabilities of 3-year, 5-year, and 7-year OS (Supplementary Figure 2D). Moreover, using the similar total risk score formula in the internal validation set, the AUC values exhibited by the total risk score for 3-, 5-, and 7-year survival reached 0.837, 0.803, and 0.824, respectively (Supplementary Figure 2E). The Kaplan-Meier curves of OS indicated that patients with low risk scores have substantially better OS than patients with higher risk scores (Supplementary Figure 2F).

In addition, the above validation process was carried out on the entire TARGET-ALL set (n=426) and revealed a good result (Supplementary Figure 3).

### The 8-lncRNA signature applied to adult ALL

**Figure 4:**
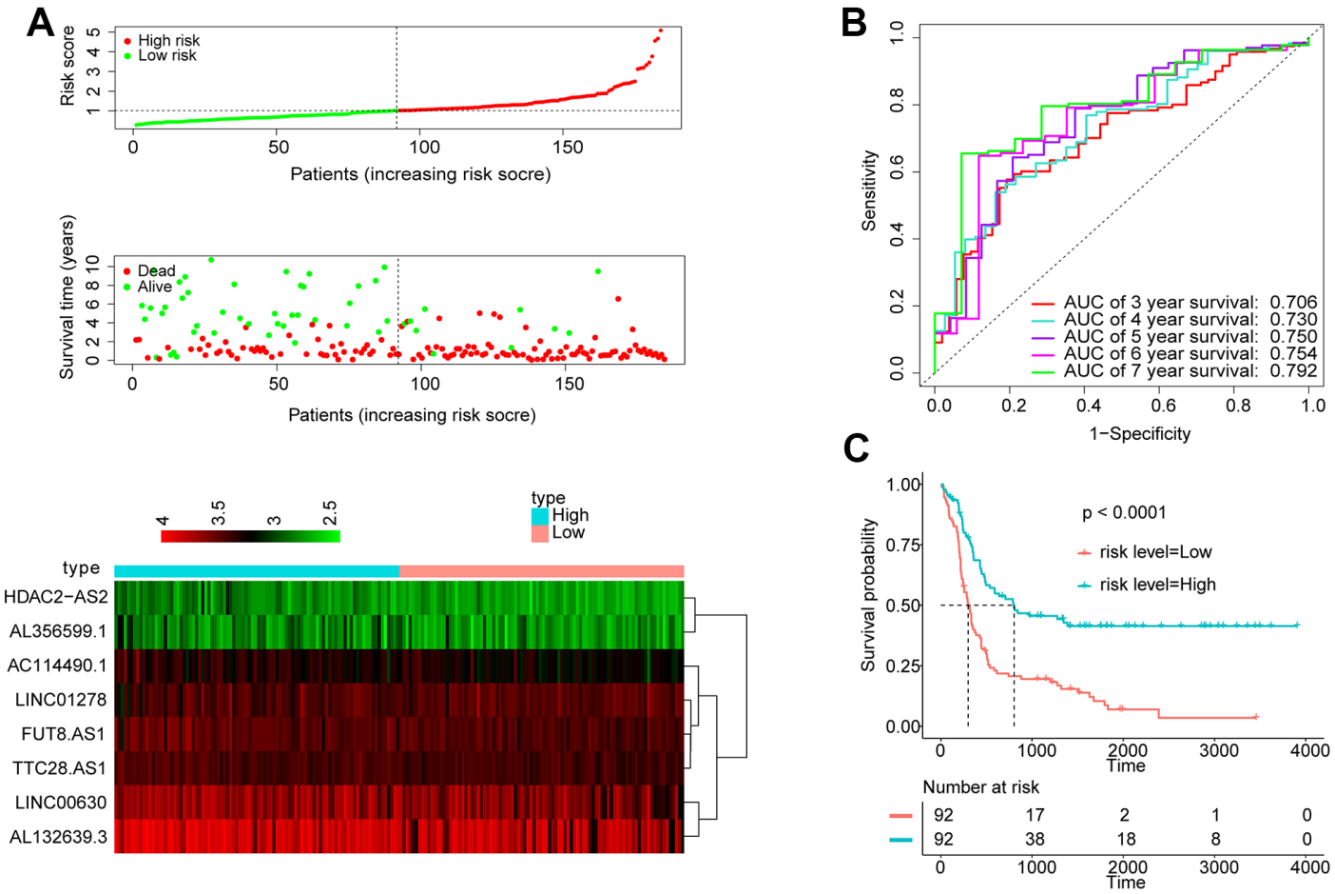
**Apply the 8-lncRNA signature to adult ALL.** (**A**) Distribution of 8-lncRNA-based signature scores, lncRNA expression levels and patient survival durations in the GSE34861 set. (**B**) ROC curve analyses based on the 8-lncRNA signature. (**C**) Kaplan-Meier curves of OS based on the 8-lncRNA signature.

To explore whether the 8-lncRNA signatures are equally applicable to adult ALL, we conducted the analysis process in the GSE34861 cohort. Taking the median score as the dividing point, participants were separated into two groups: high signature group (n = 92) and low signature group (n = 92). (Figure 4A). The AUC exhibited by the signature for 5- and 7-year survival reached 0.750 and 0.792, respectively (Figure 4B). The Kaplan Meier OS curve suggested that the OS of the patients in the low risk group was obviously better than that in the high risk group. (Figure 4C). The above indicated that this signature can also measure the prognosis of adult ALL in effective.

### Weighted gene co-expression network construction (WGCNA)

**Figure 5:**
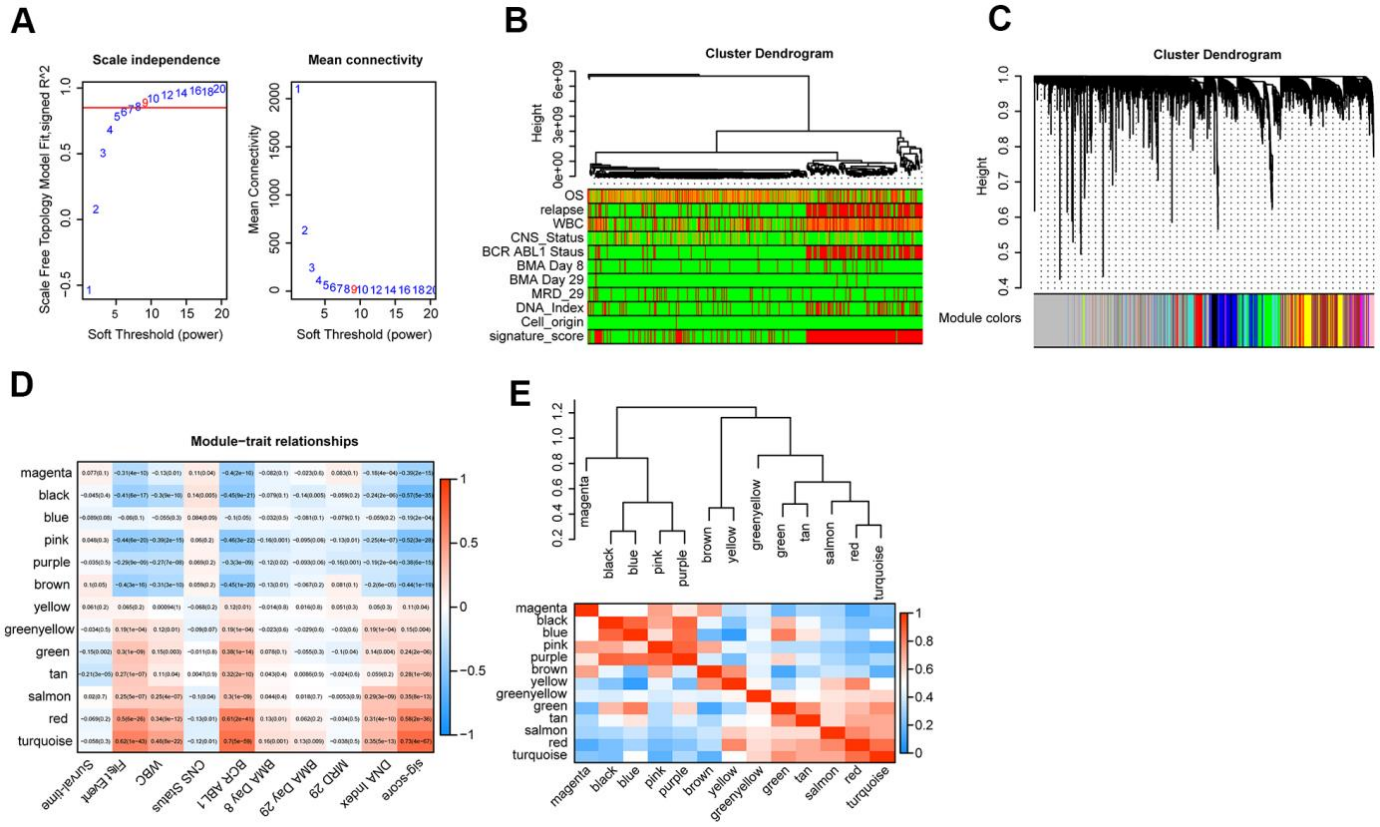
**WGCNA analysis.** (**A**) Analysis of the scale-free topology model fit index for various soft-thresholding powers (β) and the mean connectivity for various soft-thresholding powers. Overall, 9 was the best fitting power value. (**B**) Dendrogram of the genes and different clinical factors of cALL (survival time, WBC count at diagnosis, CNS status at diagnosis, BMA Blasts day 8, BMA Blasts day 29, BCR ABL1 status, MRD on day 29, DNA index, signature score). (**C**) Dendrogram of the gene modules based on a dissimilarity measure. The branches of the cluster dendrogram correspond to the different gene modules. Each leaf on the cluster dendrogram corresponds to a gene. (**D**) Module-trait relationships. Heatmap of the correlation between module eigengenes and clinical characteristics of cALL. (**E**) Hierarchical clustering and heatmap of the hub gene network.

First of all, we used Pearson correlation coefficient > 0.90 and P < 0.001 as cutoff points to screen the genes co-expressed with lncRNA. Then, we used the exp matrix composed of the mentioned 8 lncRNAs and 4985 genes as the input files. To create a scale-free system, the soft threshold beta to 9 was set (Figure 5A). Genes with similar patterns were set in different modules through average linked clustering (Figure 5B). We calculated the module eigengenes (ME) and clustered them according to their correlation to explore the co-expression similarity of all modules (Figure 5C). To identify the relationship between gene modules and clinical factors, genes were calculated to assess the correlation with clinical factors. Finally, we determined the strong correlation among gene significance and grade and signature score (Figure 5D). The 13 modules were generally separated into two clusters (Figure 5E).

### Identification of hub lncRNAs in modules and function annotation

**Figure 6:**
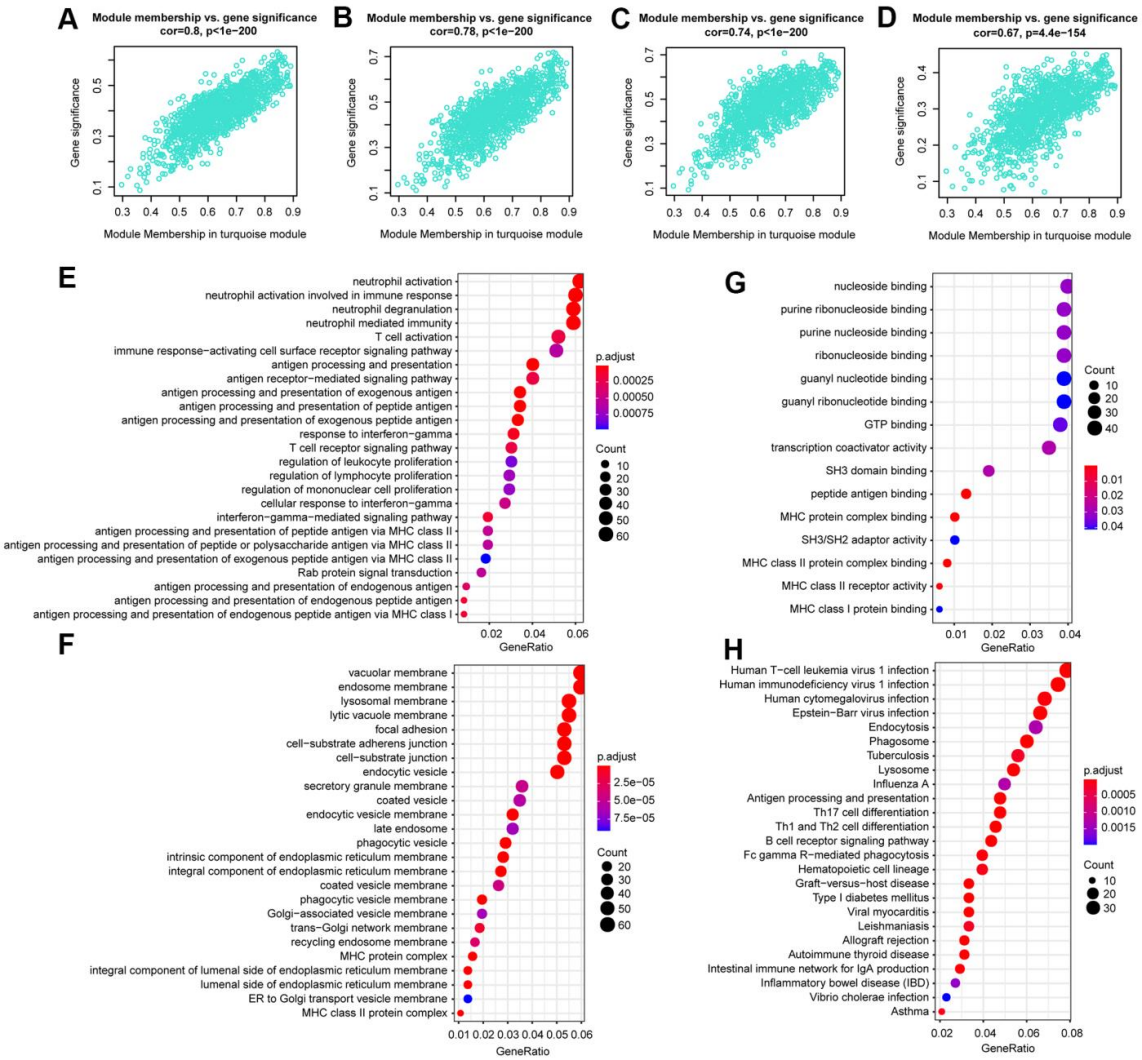
**The correlation between hub lncRNA-related genes in the turquoise module and clinical characteristics.** Correlation between the turquoise module and factors including first event (**A**), BCR ABL1 status (**B**), signature score (**C**), WBC count at diagnosis (**D**). GO and KEGG pathway enrichment of genes in the turquoise module. GO enrichment contains three categories: biological process (**E**), cellular component (**F**), and molecular function (**G**). KEGG pathway enrichment analysis revealed lymphocyte proliferation- and differentiation-related terms (**H**).

After analysis, we found that LINC01278, AC114490.1, AL132639.3, and TTC28.AS1 were all in the turquoise module, which had a strong positive correlation with first event of relapse (cor =0.8, P <1e − 200), BCR ABL1 status (cor =0.78, P <1e − 200), signature score (cor =0.74, P < 1e − 200), and WBC count at diagnosis (cor =0.67, P=4.4e − 154) (Figure 6A–6D). Furthermore, functional enrichment analysis was carried out to analyse the GO database terms and KEGG pathway associated with genes of the turquoise module (Figure 6E–6H). Finally, in the turquoise module, we generated a diagram of the mRNA-lncRNA network (weight > 0.1) of hub lncRNAs (Supplementary Figure 4).

Additionally, LINC00630, HDAC2-AS2, and AL356599.1 were together in the brown module, which had a positive correlation with WBC count at diagnosis (cor =0.4, P=3.7e − 45), first event of relapse (cor =0.37, P =12.1e − 38), BCR ABL1 status (cor =0.35, P = 2.8e − 34), and survival time (cor =0.31, P = 7e − 27) (Supplementary Figure 5A–5D). Functional enrichment analysis was also performed in the brown module (Supplementary Figure 5E–5H). Similarly, in the brown module, we constructed a diagram of the mRNA-lncRNA network (weight > 0.1) of hub lncRNAs (Supplementary Figure 5I).

### LINC01278 inhibited the apoptosis of cALL cells through hsa-miR-500b-3p *in vitro*

**Figure 7:**
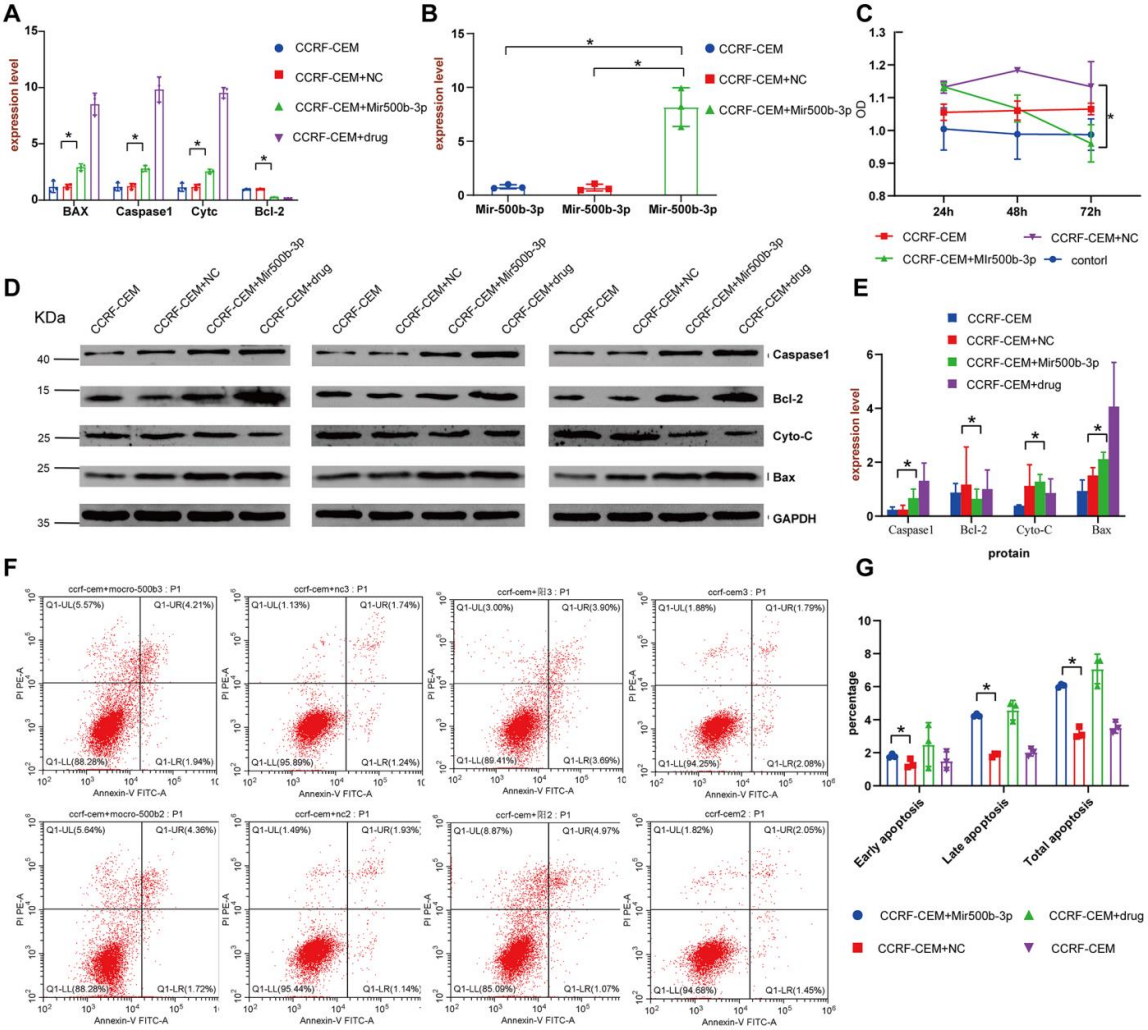
**LINC01278 affects apoptosis in CCRF-CEM cells.** (**A**) The BAX, Caspase1, Cytc and Bcl-2 mRNA expression of the CCRF-CEM cell model with LINC01278 knock down. (**B**) The miR-500b-3p expression of the CCRF-CEM cell model with LINC01278 knock down. (**C**) The proliferation of the CCRF-CEM cell model with LINC01278 knock down. (**D**, **E**) The protein expression of BAX, Caspase1, Cytc and Bcl-2 in the CCRF-CEM cell model with LINC01278 knock down. NC indicates normal control; drug indicates a control group that was exposed to a drug. (**F**, **G**) The apoptosis of the CCRF-CEM cell model with LINC01278 control group with positive drugs down; *P<0.05.

To further clarify the mechanism of how the 8 lncRNAs affect the survival of cALL patients, LINC01278 was chosen to study the mechanism using *in vitro* experiments, because WGCNA suggested that it was closely related to the expression of multiple oncogenes, and enrichment analysis showed that the genes significantly related to LINC01278 were enriched in lymphocyte proliferation and differentiation. Predicted by online websites such as starBase2.0 [21] (http://starbase.sysu.edu.cn/) and miRcode (http://www.mircode.org/), we found multiple binding sites between LINC01278 and miR-500b-3p, which suggests that LINC01278 may play a role by acting on miR-500b-3p. In the CCRF-CEM cell model with LINC01278 knocked down, we found that the well-known apoptosis-promoting molecules BAX, Caspase1, and Cytc were more highly expressed in the group with miR-500b-3p than the NC group according to qRT-PCR and Western blot assays (Figure 7A, 7D, 7E, P<0.05). However, the expression of Bcl-2, a classic inhibitor of apoptosis, was significantly decreased. Moreover, CCK8 experiments suggested that, when taking the normal control group into consideration, transfection of miR-500b-3p inhibited the proliferation of CCRF-CEM cells (Figure 7C, *P*<0.05). Additionally, after the transfection of miR-500b-3p, flow cytometry analysis indicated that the fractions of cells in early apoptosis late apoptosis and total apoptosis were significantly higher (Figure 7F, 7G, *P*<0.05). These results suggested that LINC01278 may inhibit apoptosis and increase the proliferation of cALL cells via miR-500b-3p.

## DISCUSSION

ALL is the commonest cancer in children, accounting for about 1/4 of all cancers in people under the age of 15. [22]. The cure rate of ALL is rising [4]. However, about 15 to 25 percent of patients relapse after recovery, causing death in cALL children [16, 23].

Many factors have been recorded to be associated with the prognosis of cALL, such as BCR-ABL1 fusion status [24], MRD status in the bone marrow on day 29 [25], BMA Blasts Day 29 [26], DNA Index [27] and WBC count at diagnosis [28–30]. However, there are no studies on using above factors as prognostic nomogram to foresee the prognosis of cALL. Here we figured out that lncRNAs remarkably correlated with prognosis by univariate Cox regression and LASSO analysis. Eight lncRNAs were used for construction of prognostic signature for cALL. A robust nomogram consisting of the 8-lncRNA signature, BCR ABL1 status, and WBC count at diagnosis was constructed for predicting the prognosis of cALL [31]. The AUC value of the nomogram was better than each of BCR ABL1 status and WBC count at diagnosis at 3, 5, and 7 years. The C-index value of the nomogram performs very well. The results were validated in the internal validation set and the entire validation set. Eventually, we analyzed the 8-lncRNA signature in the GSE34861 dataset and found that it could be used as a prognostic marker for adult ALL.

After a literature review, we found that two of the 8 lncRNAs, LINC00630 and LINC01278, have been studied previously. Long intergenic non-protein coding RNA 630 (LINC00630) is ubiquitously expressed in bone marrow, lymph nodes and 25 other tissues and is considered a biomarker for various cancers, such as non-small-cell lung cancer [32] and colorectal cancer [33]. In this study, LINC00630 expression was negatively linked to the risk score of cALL, and GO/KEGG pathway analysis of LINC00630-related genes in the brown module indicated that LINC00630 may regulate the occurrence and recurrence of ALL by regulating the expression of mRNAs involved in the cell cycle. Long intergenic non-protein coding RNA 1278 (LINC01278) is widely expressed in human tissue and is considered a lncRNA marker for osteosarcoma [34] and papillary thyroid carcinoma [35]. Consistent with previously published papers, LINC01278 expression positively correlated with the risk score of cALL children. Moreover, gene function enrichment analysis of LINC01278-related genes in the turquoise module indicated that it may be involved in lymphocyte proliferation and differentiation. Of note, cALL children with high LINC01278 expression appeared to have a bad prognosis.

Huang et al. [36] identified that LINC01278 downregulation decreased migration and invasion of HCC cells induced by β-catenin and TGF-β1 *in vitro* and *in vivo*. Xi et al. [37] demonstrated that LncRNA LINC01278 accelerates colorectal cancer progression via the miR-134-5p/KDM2A axis. However, in our study, *in vivo* experiments showed that LINC01278 can enhance the proliferation of leukemic cells and inhibit leukemic cell apoptosis by the inhibition of miR-500b-3p. Our study reveals for the first time the role of LINC01278 in cALL, and LINC01278 may be a biological target for the treatment of cALL in the future. LncRNA-based signatures are a novel tool that provides simple and accurate predictions of clinical results.

Nomograms have been generated for most cancer types. In the prognosis evaluation of various cancers, nomogram is in higher rate of accurate and convenient than the normal staging system [38–40]. Therefore, the use of nomograms is promoted as an option or a novel standard [41–43]. Here a prognostic nomogram of the combination of lncRNA signature and clinical factors was set. Clinical factors in nomogram cannot be influenced by investigators and are readily available. In addition, the prediction accuracy of nomogram is more reliable than each factor alone.

In conclusion, the model we have developed can firmly and predict the prognosis of patients in accurate. Our work also reveals that LINC01278 can enhance the proliferation of leukemic cells and inhibit leukemic cell apoptosis by targeting inhibition of miR-500b-3p in cALL, and LINC01278 may be a biological target for the treatment of cALL in the future.

## MATERIALS AND METHODS

### Data collection

LncRNA and mRNA RNAseq data and clinical parameters associated with cALL patients were from the NCI TARGET database (https://ocg.cancer.gov/) and GEO (https://www.ncbi.nlm.nih.gov/gds/) database.

### Cell lines and culture conditions [44]

The human CCRF-CEM cell line was from the Typical Culture Preservation Commission Cell Bank, Chinese Academy of Sciences. CCRF-CEM is a cALL cell line in the ATCC cell bank (ATCC number, CCL-119^TM^). All cells were kept at 37° C in 5% CO_2_ in MCO-18AC (PHC, Tokyo, Japan) supplemented with 1% penicillin-streptomycin (HyClone, LA, USA) solution including DMEM (GIBCO, NY, USA) and 10% FBS (EVERY GREEN, Huzhou, China).

### lncRNA knockdown and overexpression

Small interfering RNAs (siRNAs) of LINC01278 were constructed by GenePharma (Shanghai, China). Cells were transfected via Lipofectamine 2000 (Invitrogen, CA, USA). After 48 h of siRNA knockdown transfection, expression was analyzed by qRT-PCR.

### Real-time quantitative reverse transcription polymerase chain reaction (qRT-PCR) [45]

The methods of RNA extraction, reverse transcription and amplification follow those in our previous study [39]. Supplementary Table 1 presented the primers.

### Western blot analysis

Protein from cells was extracted by rotor and radio immunoprecipitation assay (RIPA) lysis buffer. Equal amounts of protein were divided by SDS-PAGE in a 12% gel and transferred to a nitrocellulose membrane. The proteins were analyzed by an optimized chemiluminescence system based on the manufacturer's instructions. We incubated the membranes overnight with the primary antibodies: anti-Caspase 1 (Servicebio, Wuhan, China, GB11383); anti-CytoC (Servicebio, Wuhan, China, GB11080); anti-Bax (Servicebio, Wuhan, China, GB11690); anti-GAPDH (Affinity, Changzhou, China, AF7021); and anti-Bcl-2 (Affinity, Changzhou, China, AFfirm063, BF9103).

### Apoptosis assay

FITC-labeled annexin V staining (Keygen, Nanjing, China) was used to determine phosphatidylserine externalization to indicate early apoptosis according to the manufacturer’s instructions.

### Proliferation assay

CCRF-CEM cells in the logarithmic growth phase were seeded in 96-well microplates with 1 × 10^4^ cells per well. CCRF-CEM cell proliferation was evaluated via the CCK-8 assay. After 24, 48, and 72 h, 10 μL of CCK-8 reagent (Dojindo Molecular Technologies, Kunamoto, Japan) was used for the cells and then the cells would be incubated at 37° C for 1 h. An automatic microtiter plate reader was set to zero bin accordance with the control wells. The absorbance (A) of each well was assessed at a wavelength of 450 nm.

### Module function annotation

The enrichment analysis of GO and KEGG functions was realized through the package org.Hs.eg.db and cluster Profiler in R software.

### Data availability

Since the datasets (TARGET and GEO) are available in public, ethical approval was not required.

## Supplementary Material

Supplementary Figures

Supplementary Table 1
