# Supplementary material for: Identification of a nomogram based on an 8-lncRNA signature as a novel diagnostic biomarker for childhood acute lymphoblastic leukemia
Source: Aging (Albany NY). 2021 Jun 9;13(11):15548–68. doi: 10.18632/aging.203116 (PMC8221355; doi:10.18632/aging.203116)
Supplement: Supplementary Figures [file aging-13-203116-s001.pdf]

SUPPLEMENTARY FIGURES

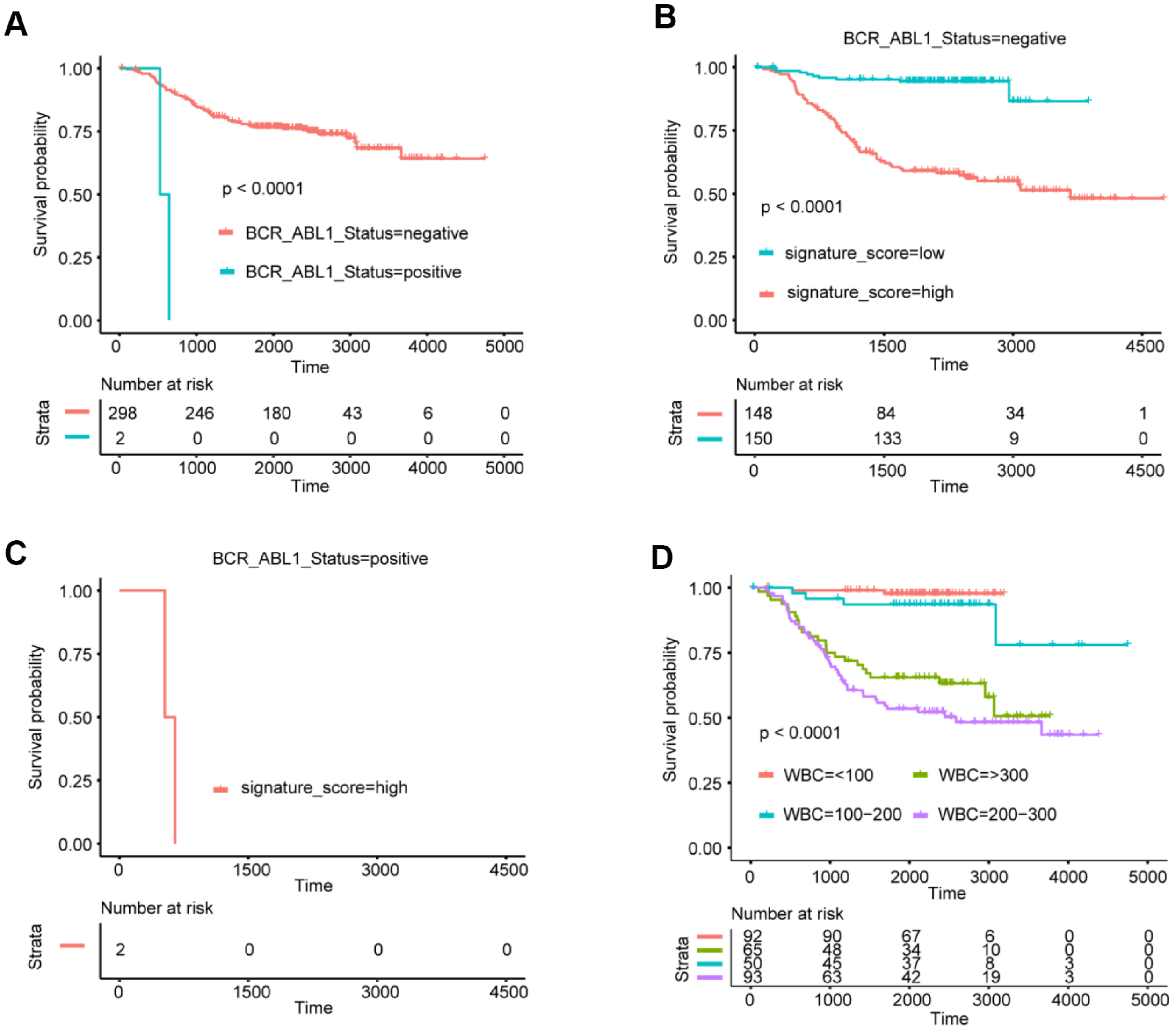

Supplementary Figure 1. Survival analysis for subgroups according to variable in nomogram. (**A**–**C**) Kaplan-Meier curves of BCR ABL1 fusion status and comparison of the survival outcomes between the high-risk and low-risk groups in different BCR ABL1 fusion gene status. (**D**) Kaplan-Meier curves based on WBC count at diagnosis, which were divided into 4 groups: "<100", "100-200", "200-300", and "> 300" according to the WBC count (*109/L).

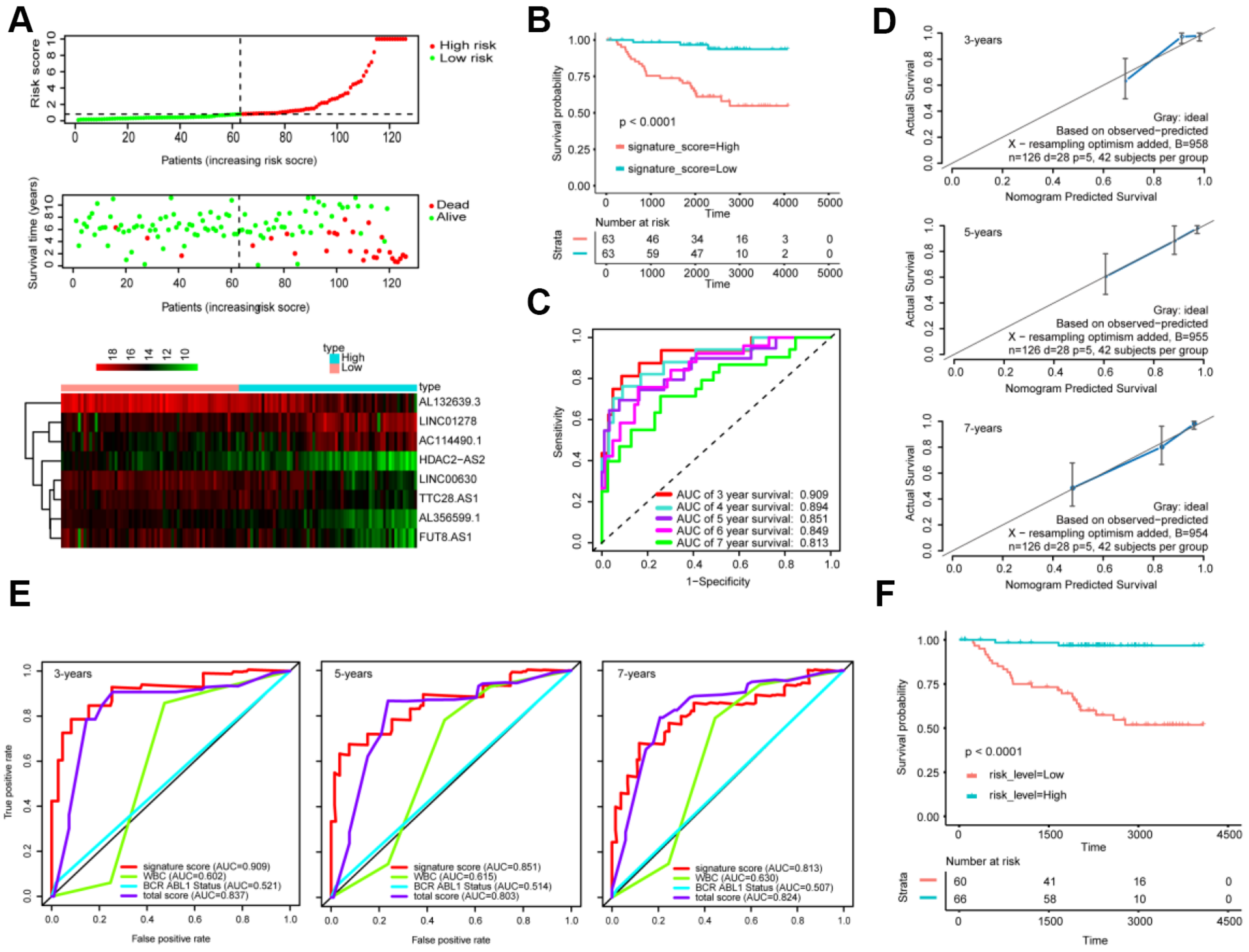

Supplementary Figure 2. Validation of the model with the internal validation set (n=126). (**A**) Distribution of 8-lncRNA-based signature scores, lncRNA expression levels and patient survival durations in the internal validation set. (**B**) Kaplan-Meier curves of OS based on the 8-lncRNA signature. (**C**) ROC curve analyses based on the 8-lncRNA signature. (**D**) Calibration curves of the nomogram for the estimation of survival rates at 3, 5, and 7 years. (**E**) ROC curves according to the nomogram and lncRNA signature score. (**F**) Kaplan-Meier curves of OS according to the total risk score.

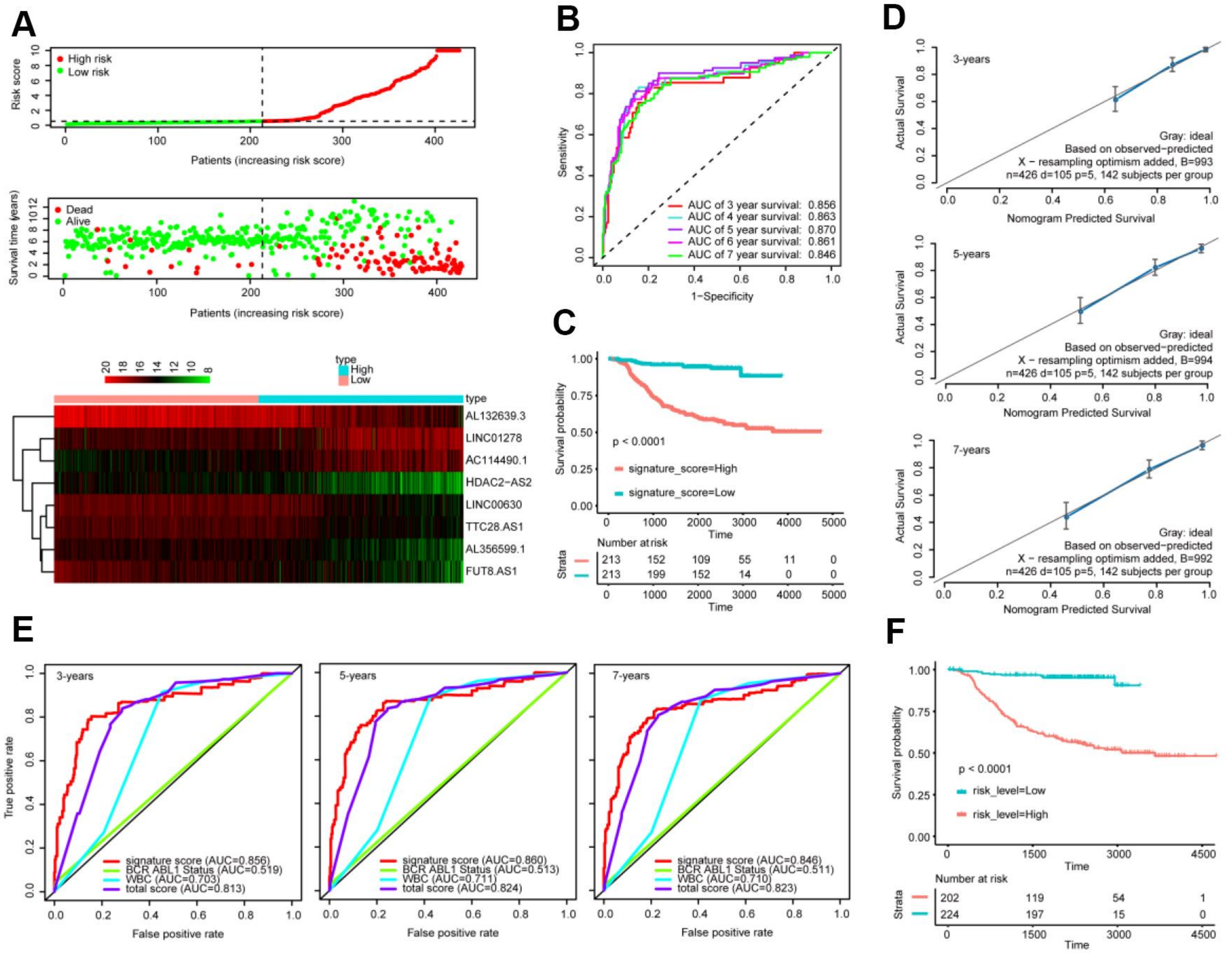

Supplementary Figure 3. Validation of the model with the entire dataset (n=426). (**A**) Distribution of 8-lncRNA-based signature scores, lncRNA expression levels and patient survival durations in the entire set. (**B**) ROC curve analyses based on the 8-lncRNA signature. (**C**) Kaplan-Meier curves of OS based on the 8-lncRNA signature. (**D**) Calibration curves of the nomogram for the estimation of survival rates at 3, 5, and 7 years. (**E**) ROC curves according to the nomogram and lncRNA signature score. (**F**) Kaplan-Meier curves of OS according to the total risk score.

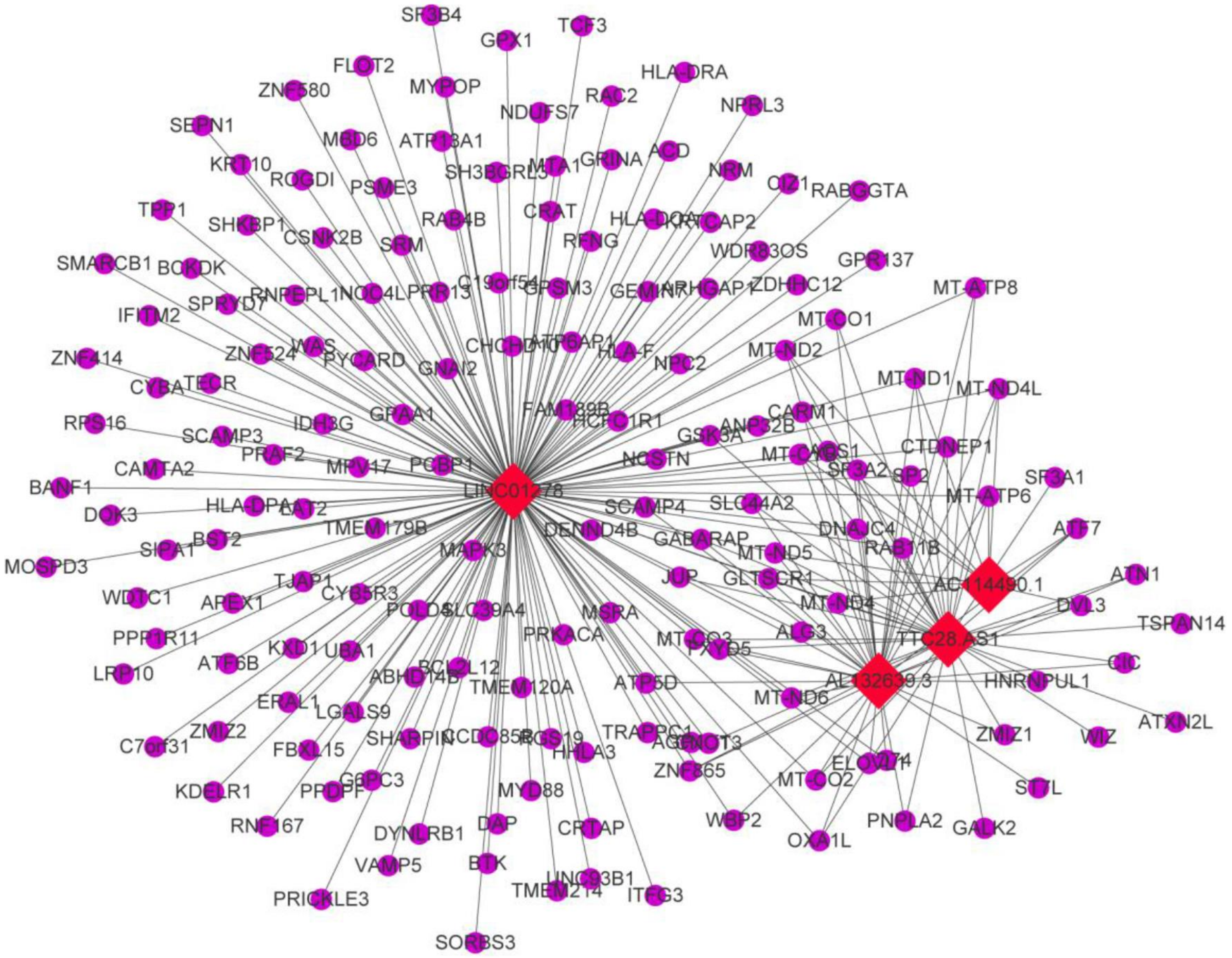

Supplementary Figure 4. The lncRNA-mRNA network based on the hub lncRNAs in the turquoise module. The red diamond shape represents lncRNAs, and purple circles represent mRNAs.

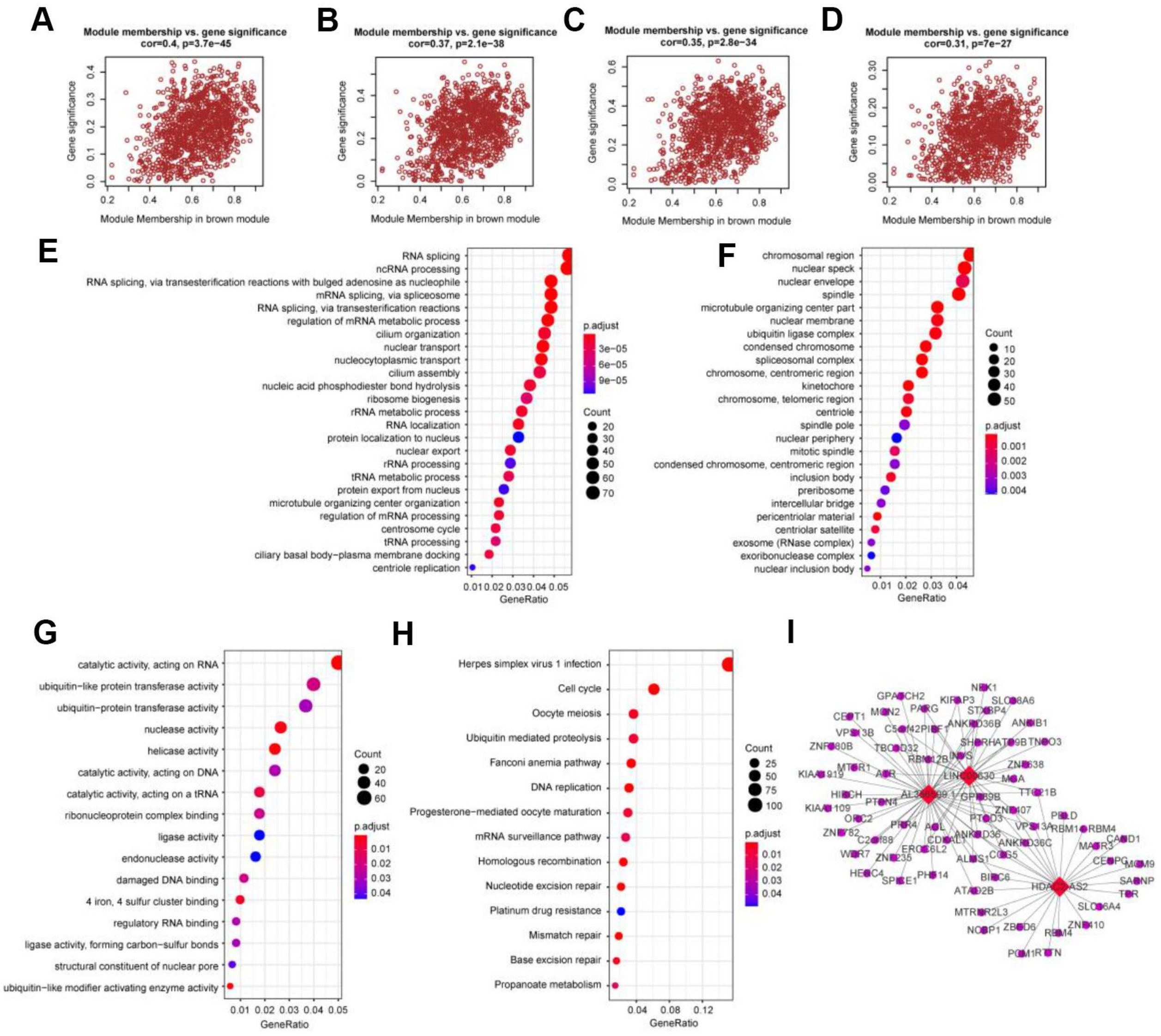

Supplementary Figure 5. The correlation between hub lncRNA-related genes in the brown module and clinical characteristics. Correlation between the brown module and factors including WBC count at diagnosis (**A**), first event (**B**), BCR ABL1 status (**C**), and survival time (**D**). GO and KEGG pathway enrichment of genes in the brown module. GO enrichment contains three categories: biological process (**E**), cellular component (**F**), and molecular function (**G**). KEGG pathway enrichment analysis revealed cell cycle-related terms (**H**). We also constructed the lncRNA-mRNA network based on the hub lncRNAs in the brown module (**I**).
