# Supplementary material for: Identification of a nomogram based on an 8-lncRNA signature as a novel diagnostic biomarker for childhood acute lymphoblastic leukemia
Source: Aging (Albany NY). 2021 Jun 9;13(11):15548–68. doi: 10.18632/aging.203116 (PMC8221355; doi:10.18632/aging.203116)
Supplement: Supplementary Table 1 [file aging-13-203116-s002.pdf]

SUPPLEMENTARY TABLE

Supplementary Table 1. Primers used in the study.

Gene	Sequence	Product length (bp)
**H-Actin**	5’-CGGCACCACCATGTACCCTG-3’<br>5’-GCCGGACTCGTCATACTCCT-3’	196
**H-Bcl2**	5’-CGACTTCTCCCGCCGCTACCG-3’<br>5’-CCCAGTTCACCCCGTCCCT-3’	128
**H-Bax**	5’-ATGATTGCCGCCGTGGACA-3’<br>5’-CCCAGTTGAAGTTGCCGTCAG-3’	88
**H- Caspase1**	5’-GAAGAAACACTCTGAGCAAGTC-3’<br>GATGATGATCACCTTCGGTTTG-3’	112
**H-cyt-c**	5’-CTTTGGGCGGAAGACAGGTC-3’<br>5’-TTATTGGCGGCTGTGTAAGAG-3’	54
